# Plant Insecticidal Toxins in Ecological Networks

**DOI:** 10.3390/toxins4040228

**Published:** 2012-04-10

**Authors:** Sébastien Ibanez, Christiane Gallet, Laurence Després

**Affiliations:** 1 Swiss Federal Research Institute WSL, Community Ecology Unit, via Belsoggiorno 22, 6500 Bellinzona, Switzerland; Email: sebastien.ibanez@wsl.ch; 2 Laboratoire d’Ecologie Alpine UMR CNRS 5553 Université de Savoie F-73376, Le Bourget-du-lac, France; Email: christiane.gallet@univ-savoie.fr; 3 Laboratoire d’Ecologie Alpine UMR CNRS 5553 Université Joseph Fourier B.P.53, 38041 Grenoble CEDEX 9, France

**Keywords:** secondary metabolism, repellent, antagonism, mutualism, pollination, coevolution, evolutionary arms race, inter-guild interactions, predators, symbionts, toxic nectar, multitrophic interactions

## Abstract

Plant secondary metabolites play a key role in plant-insect interactions, whether constitutive or induced, C- or N-based. Anti-herbivore defences against insects can act as repellents, deterrents, growth inhibitors or cause direct mortality. In turn, insects have evolved a variety of strategies to act against plant toxins, e.g., avoidance, excretion, sequestration and degradation of the toxin, eventually leading to a co-evolutionary arms race between insects and plants and to co-diversification. Anti-herbivore defences also negatively impact mutualistic partners, possibly leading to an ecological cost of toxin production. However, in other cases toxins can also be used by plants involved in mutualistic interactions to exclude inadequate partners and to modify the cost/benefit ratio of mutualism to their advantage. When considering the whole community, toxins have an effect at many trophic levels. Aposematic insects sequester toxins to defend themselves against predators. Depending on the ecological context, toxins can either increase insects’ vulnerability to parasitoids and entomopathogens or protect them, eventually leading to self-medication. We conclude that studying the community-level impacts of plant toxins can provide new insights into the synthesis between community and evolutionary ecology.

## 1. Introduction: The Diversity of Plant Insecticidal Toxins

### 1.1. Secondary Metabolites and Plant Defences

Plants have evolved a whole arsenal of defence strategies against herbivores, including the synthesis of a tremendous variety of chemical compounds. Chemical defence products may range from low molecular weight compounds, called secondary metabolites, to peptides and proteins that are active against insects (Table 1). Secondary metabolites are organic compounds that are not directly involved in the normal growth, development, or reproduction of plants [1]. We have limited our review to secondary metabolites, excluding large proteins such as lectins which can sometimes act as toxins against insects, but which are also involved in the growth and development of the plant.

**Table 1:** Examples of plant secondary metabolites with insecticidal activity.

Plant Insecticidal Compounds	Activity	Plant localization	Insect	References
**C Based compounds**				
**Terpenoids**				
Monoterpene alcohol	repellent	flowers	*Lasiusniger* (Hymenoptera)	[2]
Diterpenoids	repellentantifeeding	stems	*Ostrinia nubilalis* (Lepidoptera)	[3]
Cardenolides	toxicity	aerial and subterranean parts	*Danaus plexippus* (Lepidoptera)	[4]
Iridoid glycosides	toxicity	leavesnectar	*Junonia coenia * (Lepidoptera)	[5,6]
**Phenolics of low molecular weight**				
Phenolic glucosides	deterrenttoxicity	aerial parts	Generalist and specialist invertebrates	[7]
Aromatic esters	repellent	nectar	*Solenopsis xyloni* (Hymenoptera)	[8]
Flavonoids	repellent	leaves	*Spodoptera exigua* (Lepidoptera)	[9]
Isoflavones	feeding deterrent	roots	*Costelytra zealandicator* (Coleoptera)	[10]
Furanocoumarins and coumarins	toxicity	leaves	*Trichoplusia ni* (Lepidoptera)	[11]
Tannins	toxicity (oxidation)	leaves	*Orgyia leucostigma* (Lepidoptera)	[12]
**N Based compounds**				
Cyanogenic glucosides	toxicity	leaves	*Spodoptera frugiperda* (Lepidoptera)	[13]
Glucosinolates	toxicity	leaves	*Pieris brassicae* (Lepidoptera)	[14]
Alkaloïds	repellent	nectar	Bee pollinators	[8,15,16]
Pyrrolizidine alkaloids	toxicity	leaves	Non adapted Arctiidae (Lepidoptera)	[17,18]
Azoglucosides	toxicity (mutagen)	leaves, seeds, cones	*Rhopalotria sp.* (Coleoptera)	[19]
Non protein amino-acid	toxicity	leaves	Invertebrates	[20]
Protease inhibitors	toxicity	leaves	*Spodoptera littoralis* (Lepidoptera)	[21]
Peptides (cyclotides)	toxicity	leaves, flowers, stems, roots	Invertebrates	[22]

The insecticidal properties of these compounds (hereafter called “plant toxins”) are diverse: they may act as repellents or feeding deterrents, or induce direct toxicity leading to symptoms ranging from the inhibition of larvae or insect growth to death. In some cases, recent advances in molecular biology have made it possible to accurately identify the cellular or molecular targets of these toxins. Another active field of investigation is the elucidation of the plastic nature of such defence responses, modulated both by phytophagous (and pathogens) attacks and abiotic factors including nutrient availability [23], light [24] and drought [25]. Some of these compounds are always synthesised in the plant (constitutive resistance) while others are produced only after initial damage (induced resistance [26]). Induced defences rely on mobile metabolites with a relatively low molecular weight produced at a low cost only in the event of insect attack [27,28]. Such compounds often contain one or more nitrogen atoms, and their biosynthetic pathways derive from those of proteic amino-acids, with a potential trade off between the production of these N-containing metabolites and plant growth. Conversely, constitutive defences rely on carbon based metabolites, such as terpenoids and polyphenols that can achieve a high proportion of dry matter content in the plant and accumulate in specialised structures or compartments, such as the resin canals in the xylem of coniferous trees [23]. In such cases, production and storage costs will be high and may also compete with energy and nutrient allocation for growth and differentiation. 

Both constitutive and induced resistance have been shown to generate costs, described as allocation costs, resource-based trade offs between resistance and fitness, or as ecological costs, decreases in fitness resulting from interactions with other species (reviewed in [29], and see Section 3.3 below). Where there is limited nutrient availability plants may accumulate secondary metabolites (see the review [30]). Two theories may explain this phenomenon. The Growth-Differentiation Balance Hypothesis (GDBH) predicts that in nutrient-limited environments, plant growth is limited due to N deficiency, whilst carbon fixation produces a large surplus of carbohydrates, leading to the constitutive production of carbon based secondary compounds [31]. The Optimal Defence Theory (ODT) revolves around the idea that the tissues most vulnerable to herbivores and most valuable to the plant should be the best defended. This means that in a nutrient-limited environment, the costs of replacing damaged tissues are greater, and that plants in this environment will be constitutively well protected. Recent studies on the dominant glucosinolate defence metabolite in *Brassica *sp. confirmed the latter hypothesis, but only when the plant was not in competition with neighbouring plants [32]. The prediction that roots are less vulnerable than aerial parts and are therefore less well defended was challenged by numerous recent studies showing that defence compounds are equally distributed throughout the roots and shoots (reviewed in [33]).

### 1.2. Diversity of Effects on Insects

#### 1.2.1. Repellent Effect

Field and laboratory observations of insect attraction *vs.* repellence by plant odors have led to pioneering studies in the domain of plant-insect interactions, and current investigations are now focusing on identifying the numerous volatiles emitted by plant flowers or other organs and their additive and synergistic effects. Using genetically modified plants without scent bouquets, linalool, a monoterpene alcohol emitted by flowers of *Phlox paniculata* has been identified as the major repellent against the ant *Nasius niger* [2]. Repellence can be mediated by volatile compounds but also by non-volatile compounds acting after close contact with the insect and the plant. When different flavones identified in the invasive shrub *Lonicera maackii* were proffered to the generalist insect *Spodoptera exigua*, only apigenin appeared effective in deterring feeding whilst the related flavone luteolin had no effect [9]. The consequence of the repellent effect of the plant compounds is a modification in the insects’ foraging behaviour. The insects’ avoidance of a certain plant or group of plants producing repellent compounds creates a competitor-free niche for some insects which tolerate, or even prefer these compounds. For instance, by studying simultaneously biochemical composition (glucosinolates) and herbivory predation in twelve natural populations of the wild cabbage *Brassica oleracea*, Newton *et al.* [34] have shown that the aphid *Brevicoryne brassicae* consistently preferred plants producing progoitrin. At population level, protogoin production positively correlated with *B. brassicae *infection whilst sinigrin production negatively correlated with damage inflicted by the whitefly *Aleyrodes proletella*. Differential selection by herbivores influences the maintenance of large inter and intra-population variation in glucosinolate chemotypes in natural wild cabbage populations.

#### 1.2.2. Growth Inhibitor, Toxic Effects

Many plant compounds act as insect growth inhibitors and depending on the dose ingested, their effect can range from delayed development to a substantial reduction in fecundity and death. For example, the ingestion of luteolin flavone caused severe deleterious effects on *S. exigua* caterpillars [35], although it did not induce changes in their feeding activity in a previous study [9]. A 2 g/L concentration induced 43% mortality after 11 days, and surviving larvae were 50% lighter than the control larvae. Moreover, rates of both pupation and emergence were lower compared to the control. This type of serious negative impact of plant toxins at all insect ontogenetic stages reduces the population growth rate and select for insects capable of overcoming the plant’s defence (*i.e*., specialisation). 

#### 1.2.3. Pleitropic Role of Tannins

The role of tannins as plant defence compounds, due to their ability to complex proteins, has been extensively investigated, but contrary to early theories [36] and to what happens in vertebrate herbivores, tannins seem to have no effect on protein digestion in insect herbivores [37]. Recent works [12] suggest that toxicity is more likely to be due to oxidation mechanisms forming semiquinone radicals and quinones, as well as other reactive species in insect guts with high pH levels. However, it is still unclear as to whether the fatal lesions observed in the midgut, and especially in the peritrophic envelopes of the caterpillar *Orgyia leucostigma*, can be directly attributed to the tannins or to oxidative stress. It is also likely that hydrolysable tannins, whose hydrolysis is facilitated in the insect digestive tract, will act differently from the less biodegradable condensed tannins [38]. Deterrence due to tannins has also been questioned, because whilst their astringency can act as a powerful feeding deterrent for many invertebrates, some adapted species seem to benefit from small amounts of tannins in their diet (see discussion in [39]).

## 2. Insecticidal Toxins in an Antagonistic Context

### 2.1. Strategies Selected in Insects to Overcome Plant Chemical Defences

Insects have evolved multiple strategies to overcome the diverse toxins produced by plants, including contact/ingestion avoidance, excretion, sequestration, toxin degradation, and target-site insensitivity (reviewed in [40]). In the case of behavioural avoidance, plant toxins act as a deterrent and modify insect foraging behaviour. The nutrition dilution hypothesis suggests that insects may limit toxin ingestion by adopting a more general diet, meaning the toxins specific to each plant species are ingested in small amounts [41]. A widespread insect defence against plant toxins involves degrading and excreting them using a variety of metabolic means [40], sometimes with the help of a symbiotic partner [42]. Plant compounds can also be sequestered and subsequently used as a defensive substance against predators or pathogens (reviewed in [43]), such as pigments for adult coloration or pheromones (reviewed in [44]). The best known example is that of the monarch butterfly caterpillars sequestering the cardenolides produced by their host-plants (milkweeds): the plant’s chemical defence is used both at the larval and adult stages as protection against predators. Plant toxins may also play a role in sexual selection as shown in the arctic moth *Utetheisa ornatrix* [45]. Larvae sequester pyrrolidine alkaloids (PAs) from their host plants, PAs are retained by the adults and passed on by the females to the eggs: all the stages are therefore protected against predators. The male has an unusually large spermatophore containing PAs that are transmitted to the female as a nuptial gift at mating. Furthermore, males also modify PAs into courtship pheromone, and the level of pheromone produced positively correlates with the amount of PAs in the nuptial gift. Insects able to sequester host-plant toxins have a dual advantage in terms of the colonisation of a free-competitor open niche and the usurpation of the plant’s chemical defences.

### 2.2. The Co-evolutionary Arms Race and the Evolution of Specialisation

Over the last 50 years, plant defence theories have been formulated to explain the impressive variation in abundance, distribution, and diversity of plant secondary compounds and other defensive traits. Traditional theories of enemy-driven evolutionary dynamics (or antagonistic co-evolution) have hypothesised that defensive traits escalate through a co-diversification process. Applied to plant-insect interactions, this co-evolutionary arms race theory predicts a never ending race between plants innovating to produce new insecticidal toxins (and many other anti-insect weapons) and insects evolving resistance traits [46]. In this context, insecticidal toxins might act as major drivers of both plant and insect biodiversity, the two most diversified eukaryotic groups on Earth. Plant toxins select insects capable of overcoming the defence (specialists) thereby creating a competitor-free niche for these specialists. Changes in the competitive abilities of the different visitors modify the selective pressures plants are under to produce toxins. Whilst insect specialisation is a predicted outcome of co evolution, escalation towards ever-increasing toxin production is not necessarily expected in plants. Indeed, specialist insects often use plant toxins for their own protection or as pheromones, and preferentially feed on more toxic plants, thereby selecting for decreased chemical plant defences. For example, basal clades of *Aristolochia* produce aristolochic acids, which are sequestered by specialist Troidini butterflies, while derived species produce labdanoic acids which negatively affect the specialist butterflies. In milkweeds (*Asclepias*), cardenolides, which are sequestered by specialist monarch butterflies, show a phylogenetic decrease, while phenolic compounds, which are not sequestered, show a phylogenetic escalation [47]. Interestingly, plant re-growth, *i.e*., plant tolerance rather than chemical defence appears to be selected during the course of co-evolution [47]. Therefore, alternatives to the arms race escalation for plants could be to tolerate, or even to use the specialist herbivores capable of overcoming chemical defences as privileged partners. Indeed, the production of insecticidal toxins could be a way for plants to select a few specific insects to interact with, giving reciprocal benefits. 

## 3. Plant Toxins in Mutualistic Interactions

Plant toxins, by definition, affect other organisms negatively, so toxins are expected to be involved only in conflictual relationships between species. However, they are not restricted to purely antagonistic interactions and also play an important role in mutualistic interactions. Indeed, conflicts are widespread in mutualisms because interacting species tend to limit their costs and increase their benefits at the expense of their partners. Plants can use toxins to alter the balance between costs and benefits to their advantage using several mechanisms that we will describe here in detail. To date, research in this area has focused on plant-pollinator interactions, but the mechanisms also apply to other mutualisms e.g., plant-seed dispersers.

### 3.1. Plants Use Toxins to Choose Adequate Partners

Plants can exclude species that are too costly and/or that do not provide them enough benefits by means of a wide variety of barriers, including toxins. Indeed, insecticidal toxins are very often produced in nectar, pollen, and seeds or fruits [48]. Baker [49] found that the nectar of 50 out of 567 tested species contained alkaloids, and 191 out of 528 contained phenolics. These nectar toxins often act as filters that are selective for specific pollinators. For example, the nectar of the South African succulent shrub *Aloe vryheidensis *contains phenolics, whose bitter taste repels unwanted honey bees and sunbird nectarivores and whose dark colour attracts suitable frugivorous and insectivorous bird pollinators [50]. The nectar of the Carolina jessamine (*Gelsemium sempervirens*) contains the indole alkaloid gelsemine, a secondary metabolite toxic to vertebrates, which is highly distasteful and a deterrent for most pollinators [51], but which consistently attracts the bumble bees *Bombus impatiens* and *B. bimaculatus*. In both examples, the toxins simultaneously attract suitable pollinators and repel those less well suited. In the extreme case of nectar robbing, it has also been shown that toxic nectars deter thieves but not legitimate pollinators [5,52]. In such cases, when toxins are able to filter suitable partners, they constitute an equivalent to the morphological or phenological barriers evolved in specialised plants such as deep corolla tubes that prevent short-tongued generalist insects from feeding on the nectar. However, it should be noted that despite their repellent properties, putatively toxic nectars do not always alter the offspring performance of nectar-collecting bees [53]. Like herbivores, suitable mutualistic partners might have developed specialised detoxification adaptations to overcome toxic nectars and even to use them against pathogens ([54] and Section 4.2 below).

To date, most studies on toxic nectar have focused on single plant species, but the community-level consequences of toxic nectar should also be considered. Indeed, it has been shown that different experimental “ecological” contexts, such as the presence of alternative non-toxic nectar sources, influence pollinator deterrence due to alkaloids in floral nectar [15]. In natural plant communities, several co-occurring plant species are likely to produce toxic nectar, which might affect the whole network structure as well as morphological or phenological traits.

### 3.2. Plants Use Toxins to Control Mutualistic Partners

Even suitable partners are tightly controlled in order to limit the costs and maintain the benefits of mutualism. For example, a physiological control mechanism has been described in plants pollinated by insects whose larvae develop on seeds, where selective fruit abortion of over-infested fruits limits plant costs and makes it possible to control the partner [55]. Similarly, a toxin (adonivernith) produced by the globeflower *Trollius europaeus* allows the plant to limit seed predation costs inflicted by its specific pollinator’s larvae *Chiastocheta *spp. [56,57]. The evolution of the chemical defence reduced the cost of the interaction with the *Chiastocheta* flies, and thereby favoured the evolution of flower morphological specialisation for those flies [58]. In this example, toxin production stabilises the mutualistic interaction over an evolutionary time-scale. Similarly, the toxic nectar of *Nicotiana attenuata* optimises the number of flower visitors per volume of nectar produced, allowing plants to keep their nectar volumes small and thereby reduce the costs of mutualism [8], while promoting out-crossing.

Partner control through toxins can also occur at a fine mechanistic, behavioural level. For example, the cycasin toxin of cycads is more abundant in female than in male cones, forcing their herbivore pollinating beetles to concentrate in male cones, which might prevent seed predation and ensure pollen export [19]. In *Gelsemium sempervirens* toxic nectar was shown to encourage pollinators to leave plants after visiting only a few flowers, thus reducing self-pollen transfer by about half [16]. However, nectar alkaloids also reduced pollination, so that they had apparently more costs than benefits for plants [51,58]. This apparent cost of toxic nectars may be partially offset by the superiority of out crossed progeny. Toxic nectar strongly impacts on the pollination process, but it has not yet clearly been demonstrated that it gives plants an adaptative advantage. This raises the question of the evolutionary origins of toxins involved in mutualistic interactions.

### 3.3. Toxins in Mutualisms Usually Evolve in Relation to Anti-Herbivore Defence

Plant traits evolve in response to the selection pressures exerted by many agents, abiotic conditions, competitors, herbivores and mutualists. In the case of toxic secondary compounds, the major selection pressure is the presence of herbivores. Under this scenario, toxins first arise as an anti-herbivore defence and subsequently impact on the interactions with mutualists. For example, *Dalechampsia* vines first evolved toxic triterpen in resins to protect staminate flowers against pollen feeders, which later became a reward for resin-collecting bees [59,60]. In some lineages the association with resin-collecting bees was subsequently lost, and the production of resin was re-invested in the defence against florivores [61]. The resin of *Dalechampsia* vines is a “transfer” exaptation in that it is captured by the reward function at the expense of the defence function, but in other systems the toxins impact simultaneously on antagonists and mutualists. In the globeflower *Trollius europaeus*, the flavonoid adonivernith is particularly abundant in the sepals forming the globose corolla [56], which is likely to be a defence against generalist flower herbivores, and at the same time it accumulates in the carpel walls of the infested fruits to limit seed predation from its mutualistic flies [57].

Toxins linked with anti-herbivore defence sometimes impact on mutualistic interactions in a positive way for the plant, as in the two examples above, but they can also have a negative impact, as it has been hypothesised for toxic nectars, which might simply be a by-product of the presence of toxins in other parts of the plant [48]. In this case, toxic nectar might not confer any adaptive advantage with respect to pollination [50], and there would instead be a trade-off between anti-herbivore defence and pollinator attraction, *i.e*., an ecological cost of anti-herbivore defence.

## 4. Insecticidal Toxins and Multi-Trophic Interactions

Plant insecticidal toxins do not only affect insects (herbivores and pollinators), but also can have positive or negative effects on their natural enemies. Natural enemies of insects include macropredators (invertebrate and vertebrate predators) and micropredators (parasitoids and entomopathogens such as viruses, bacteria, fungi and nematodes). 

### 4.1. Effect of Plant Toxins on Predators and the Evolution of Aposematism

Many insects sequester plant insecticidal toxins that are then used as protection against predators, sometimes throughout all the ontogenetic stages (see Section 2.1 above). The sequestering stage is usually aposematic, exhibiting a strong warning signal (usually visual, but sometimes olfactory or acoustic) towards predators. Plant metabolites sequestered by insects may function as gustatory deterrents, as emetic irritants, or as deadly poisons, depending on the dose ingested [44]. The repellent taste of toxic substances associated with an aposematic signal is central to prey-predator interactions allowing the predators to avoid ingesting the toxin before injuring the prey. In many cases, the toxins are sequestered in the insect’s cuticle, promoting predator deterrence at the first contact. However, if a predator fails to detect a systemic toxin, the induction of emesis would make it possible to eliminate the toxin before a lethal dose is reached. One well known example is that of *Heliconius *butterflies from the tropics of the New World that are able to sequester cyanoglucosides from *Passiflora* host-plants to varying degrees [46]. These butterflies exhibit bright color patterns signalling their distastefulness to their predators. Furthermore, wing colour patterns between species within a given habitat are more similar than within species across localities, a phenomenon known as Mullerian mimicry. The adoption of a common aposematic signal by the local community of unpalatable insects limits the cost of predators’ learning. Depending on the *Heliconius* species and on the chemical characteristics of the *Passiflora *plants exploited, the butterflies are more or less toxic, but they all benefit from the same protective aposematic pattern. 

### 4.2. Effect of Plant Toxins on Parasitoids and Entomopathogens

Plant insecticidal toxins can have a positive impact on insect natural enemies in several ways. Insecticidal toxins may slow down insect growth rate, extending the timeframe during which the herbivore is vulnerable to parasitism or predation by its natural enemies. Toxins may also weaken host immune responses, rendering them more susceptible to parasitism. For example, the ingestion of certain plant secondary metabolites (e.g., iridoid glycosides) can diminish the immune response by directly interfering with melanisation [6], or more indirectly by reducing herbivore feeding performance [62]. In the specialist buckeye caterpillar *Junonia coenia* (Nymphalidae), which sequesters iridoid glycosides from its host plants and uses them as anti-predator defences, larvae feeding on diets with high concentrations of iridoid glycosides are more likely to have lower immune response than those feeding on diets with high concentrations of these compounds. This suggests that larvae feeding on plants with high toxin concentrations might be less well defended against parasitoids, whilst at the same time being better defended against predators [6]. 

The toxicity of the natural entomopathogen *Bacillus thuringiensis kurskaki* (Btk) for the cabbage looper *Trichoplusia ni* was shown to increase on tomato relative to cucumber and pepper leaves [63]. The chemistry of tomatoes might explain this increased toxicity as chlorogenic acid, a constitutive defensive compound in tomatoes, in combination with peroxidase, an inducible defensive compound in tomatoes, was shown to increase Btk toxicity towards another agronomic pest, the corn earworm *Heliothis zea* [64]. As Btk is widely used as a biopesticide in agronomy, this synergy observed between plant allelochemicals and the entomopathogen is of agronomical interest. However, this also means that selection for Btk resistance is higher in tomatoes compared to other host plants. Indeed, during a three-year survey of Btk resistance in greenhouses in Canada, the highest Btk resistance levels were reported in *T. ni* collected in tomato greenhouses [65]. 

On the other hand, plant insecticidal toxins also commonly have a negative impact on parasitoid/entomopathogen ﬁtness, and may compromise the use of natural enemies as pest control agents. Negative effects may occur directly, when they affect the developing larva or viral/bacterial multiplication inside the herbivore body, or indirectly, when the pathogen suffers from low host size or quality, or from enhanced insect immunity [66]. For example, the ability of *Parasemia plantaginis* (Arctiidae) caterpillars to encapsulate a foreign object varies according to the amount of antioxidant compounds (e.g., carotenoids and phenolics) present in the host plant species [66]. The strong negative impact of α-tomatine (a glycoalkaloid in tomatoes) on the fitness of the generalist endoparasitoid of noctuid larvae *Hyposoter exiguae* has been demonstrated and adding nicotine to the diet of the tobacco hornworm induced higher larval mortality in its specialist parasitoid *Cotesia congregata* (see [43] for many other examples). The generalist pollinator *Bombus impatiens* feeds on a large range of nectariferous plants, including plants producing toxic nectar; it was shown that nectar containing a high concentration of the alkaloid gelsemine negatively affected bumble bee fecundity [67], but that gelsemine ingestion by bumble bees infected with the gut protozoan *Crithidia bombi* reduced pathogen loads [45]. 

The negative impact of plant toxins was even shown to occur in a four-trophic-level interaction involving plants–herbivores–parasitoids and hyperparasitoids. The hyperparasitoid *Lysibia nana* parasitising the endoparasitoid *Cotesia glomerata,* itself infecting the caterpillar *Pieris brassicae* was found to be negatively affected by high concentrations of glucosinolates in the diet of *P. brassicae* [14]. 

Different plant compounds have been shown to have both positive and negative effects on entomoviruses [68]. For example, in the Gypsy moth *Lymantria dispar*, nuclear polyhedrosis virus (NPV) induces increased mortality when ingested with salicin (a phenolic glycoside present in aspen foliage), but decreased mortality in the presence of tannins, another common group of plant phenolic compounds [69]. 

There is overwhelming evidence that the impact of pathogens can be mitigated by the insect’s diet. The mixed diet adopted by polyphagous insects might indicate a compromise between the negative impact of some plant toxins on herbivore and their positive effects on natural enemies [70]. 

### 4.3. Plant Toxins, Pharmacophagy, and Self-Medication in Insects

Pharmacophagy is the active collection of plant substances for the purposes of protection against pathogens rather than nutrition: toxic plants are preferentially foraged even if it implies a fitness cost (e.g., slower growth or reduced fecundity), because it confers protection against predators or pathogens [71]. Several social insect species, including wood ants and honey bees, are known to collect antimicrobial plant compounds to prevent pathogen proliferation within their hives [72,73]. 

Furthermore, there is increasing evidence that infected insects change their diet foraging behaviour and actively seek plants rich in secondary metabolites inducing a lower growth rate rather than a nutrient-rich but toxin-poor plant diet (self-medication). For example, parasitoid-infested *Platyprepia* *virginalis* caterpillars preferentially consumed hemlock instead of lupine, their primary host plant, in field choice experiments; unparasitised caterpillars were more likely to survive to adulthood when feeding on lupine, whereas parasitised caterpillars were more likely to survive on poison hemlock [74]. Host-plant choice by infected insects may depend on the stage of parasitoid infection. At the late stage of infection, woolly bear caterpillars *Grammia incorrupta* (Arctiidae) improve their survival by selectively feeding on diets rich in pyrrolizidine alkaloids that confer resistance against tachinid flies, while excessive ingestion of these toxins reduces the survival of unparasitised caterpillars [17]. However, at the early infection stage, fly-infected larvae prefer feeding on a diet rich in iridoid glycosides while wasp-infected larvae prefer feeding on plants rich in antioxidants such as flavonoids [18]. Variations in the feeding behaviour of infected insects throughout ontogenetic stages might be explained by complex trade-offs between nutrient-quality requirements and immune response energetic costs. 

### 4.4. Plant Toxins and Insect Symbionts

Insects harbour a large diversity of symbionts, including gut microbiota and bacteria found inside the insects’ cells (endosymbionts). Of the many roles played by symbionts in insect biology, they have a key role in insect nutritional ecology by helping with digestion and providing nutrients that are limited or lacking in the diet (see [75] for a recent review). They are also believed to have a role in metabolising plant secondary compounds [76]. For example, the microbial flora of the desert locust gut is able to metabolise secondary plant chemicals in order to produce antimicrobial phenolics contributing to host defence against pathogens. Other plant-derived metabolites produced by the gut microbial flora are used by the host as components of the aggregation pheromone in fecal pellets [42]. Furthermore, the different enterobacteria taxa were shown to metabolise plant secondary compounds more or less efficiently, but no direct impact of plant toxins on bacterial growth was found [42]. Although many studies indicate that interactions between plants and insect symbionts have an impact on herbivores’ growth (either positive or negative), the mechanisms that drive these changes are not clear, and the role of plant toxins on the microbial symbiotic community structure of herbivores has not yet been studied and is still to be elucidated.

## 5. Conclusions

**Figure 1:**
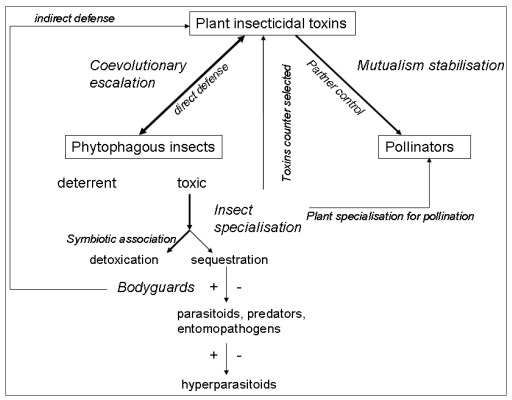
Schematic representation of the possible roles of plant insecticidal toxins in ecological networks. Arrow size represents the probable strength of the effect and the double-headed arrows indicate where co-evolution is expected. Positive and negative effects of plant toxins on higher trophic levels are indicated by + or − signs.

The ecology and evolution of plant toxins cannot be understood in a purely plant-insect context. The fitness consequences of toxin production on both plants and insects depend on its community-level consequences. Almost all trophic levels in an ecosystem can be impacted by plant toxins: plant mutualists, insect competitors, predators, pathogens and parasitoids (Figure 1). Moreover, in this review we restricted ourselves to above-ground processes, but the presence of toxins in leaves also affects below-ground processes [77]. The consideration of the whole community context explains otherwise strange insect behaviours, e.g., specialised pollinators that are attracted by toxic nectar or herbivores that select toxic foods to cure themselves. A key challenge is now to integrate the different ecological consequences of toxin production by plants across all the trophic levels impacted, in order to be able to assess the selection pressures exerted on toxin production by plants, and on strategies for dealing with toxins at all the trophic levels. Moreover, species assemblages in communities are highly variable in space and time, so toxic plants will not experience the same insect herbivores and mutualists, and insects will interact with different toxic plants. This provides an ecological basis for explaining the tremendous diversification of toxin production and strategies for dealing with toxins.

Finally, from an applied perspective, an improved understanding of the effects of the different plant toxins on pests and their natural enemies and symbionts would help to optimise integrated pest management (IPM) strategies. Optimal IPM strategies should take into account not only the target insect pest throughout its development, but also the whole ecological context, including the plant allelochemicals, and the insect’s competitors, predators, pathogens and symbionts. 
